# A Lumped-Parameter Cardiovascular Model for Investigating Hemodynamic Alterations During Atrial Fibrillation

**DOI:** 10.3390/bioengineering13060639

**Published:** 2026-05-29

**Authors:** Prashant Kishor Sharma, Yu-Chien Tung, Chia-Yuan Chen

**Affiliations:** Department of Mechanical Engineering, National Cheng Kung University, Tainan 701, Taiwan; prashant94580@gmail.com (P.K.S.); n16141010@gs.ncku.edu.tw (Y.-C.T.)

**Keywords:** atrial fibrillation, atrial remodeling, cardiovascular modeling, pressure–volume analysis, lumped parameter model

## Abstract

Atrial fibrillation (AF) is a major cardiac arrhythmia characterized by impaired hemodynamics caused by irregular ventricular activation and loss of coordinated atrial contraction. However, the coupled effects of rhythm irregularity and progressive atrial remodeling on cardiovascular hemodynamics have not been sufficiently quantified. In this study, a closed-loop lumped-parameter cardiovascular framework was developed to investigate hemodynamic alterations under normal sinus rhythm (NSR) and AF conditions. Time-varying elastance functions were used to represent cardiac chamber mechanics, while stochastic RR interval sequences reproduced the irregular ventricular response characteristic of AF. Progressive atrial remodeling was represented through systematic increases in atrial elastance to simulate increasing chamber stiffness. The results demonstrated that AF produced irregular left atrial pressure fluctuations and pronounced beat-to-beat variability in ventricular pressure and volume. Ventricular stroke volume decreased from 70–75 mL under NSR to 55–65 mL under AF conditions. With progressive remodeling, left atrial volume decreased by 36.4%, while ventricular end-diastolic volume increased from 130 to 134 mL, indicating compensatory ventricular adaptation. These findings suggested that atrial stiffening impaired atrial compliance and reservoir function, whereas ventricular adaptation partially compensated for the impairment in atrial mechanical function.

## 1. Introduction

Atrial fibrillation (AF) is one of the most prevalent cardiac arrhythmias, affecting more than 37 million individuals worldwide [1,2,3]. It is associated with a fivefold increase in stroke risk, a threefold increase in heart failure incidence, and significantly elevated cardiovascular mortality. The hemodynamic impairment observed in AF arises primarily from two interacting mechanisms: the loss of coordinated atrial contraction and the irregularity of ventricular activation [4,5,6,7,8,9,10]. Under normal sinus rhythm (NSR), atrial contraction contributes to late diastolic ventricular filling, commonly referred to as the atrial booster pump function [11,12]. In contrast, AF disrupts this coordinated contraction and introduces irregular RR intervals, leading to beat-to-beat variability in ventricular preload, stroke volume, and systolic pressure. In addition to these functional disturbances, AF is associated with progressive structural remodeling of the atrial myocardium. This remodeling is characterized by fibrosis, myocyte loss, and chamber dilation, which collectively increase atrial stiffness and reduce compliance [13,14,15]. As a result, the atrium loses its ability to function effectively as a reservoir, conduit, and contractile chamber. These structural changes further impair ventricular filling and reinforce the hemodynamic consequences of rhythm irregularity [16,17,18,19]. Therefore, AF represents a condition in which electrical, mechanical, and structural alterations interact closely, leading to progressive deterioration of cardiac performance [20]. In addition to mechanistic cardiovascular analysis, computational hemodynamic models may provide important support for biosensor and bioengineering applications. Physiological quantities predicted by lumped-parameter cardiovascular models, including pressure waveforms, flow dynamics, and rhythm variability, are closely related to signals acquired using wearable and implantable cardiovascular sensing technologies. Consequently, such modeling frameworks may assist in interpreting biosensor-derived physiological data, evaluating signal-processing approaches, and developing AF monitoring strategies.

The independent and coupled contributions of these mechanisms to hemodynamic dysfunction have not been sufficiently quantified [19,21,22]. Experimental and clinical measurements of pressure–volume relationships under irregular rhythm conditions are limited by technical constraints and patient variability [23,24]. As a result, it remains challenging to isolate the effects of rhythm irregularity from those of structural remodeling in vivo [25]. Furthermore, the dynamic interaction between atrial mechanical impairment and beat-to-beat ventricular response has not been directly measurable under controlled conditions. This limitation has restricted the ability to evaluate how these mechanisms jointly influence cardiac performance systematically [26,27]. Computational modeling has therefore emerged as an effective approach for investigating cardiovascular dynamics under controlled and reproducible conditions [28]. In particular, lumped-parameter cardiovascular models provide an efficient framework for capturing global hemodynamic interactions across cardiac chambers and vascular compartments. Previous modeling studies have investigated AF-induced hemodynamic alterations by incorporating stochastic RR interval sequences to reproduce ventricular rate irregularity or by modifying atrial contractility to represent impaired atrial function [29,30,31,32]. However, these studies have typically examined rhythm irregularity and structural remodeling in isolation, without quantifying their coupled effects on pressure–volume behavior [33,34,35]. Therefore, the relative contribution and interaction of rhythm irregularity and structural remodeling remain unclear [36,37].

To address this gap, a closed-loop lumped-parameter cardiovascular model was developed in the present study. Cardiac chamber mechanics were described using time-varying elastance functions, while stochastic RR interval sequences were implemented to reproduce the irregular ventricular response characteristic of AF. Progressive atrial structural remodeling was represented through systematic variation in atrial elastance to simulate increasing chamber stiffness and impaired atrial compliance. The present framework combined stochastic RR-interval variability and progressive atrial remodeling within a unified closed-loop cardiovascular model to investigate their coupled hemodynamic influence systematically. This formulation enabled direct evaluation of the coupled effects of rhythm irregularity and atrial mechanical impairment on pressure–volume behavior, ventricular filling dynamics, and atrioventricular coupling across different remodeling stages. Using this model, the hemodynamic differences between NSR and AF were quantified, and the effects of progressive atrial remodeling on pressure–volume relationships and ventricular filling dynamics were analyzed. The results provided mechanistic insight into atrioventricular coupling in AF, suggesting that ventricular adaptation partially compensated for impaired atrial function, while overall hemodynamic efficiency may be reduced.

## 2. Materials and Methods

**Figure 1:**
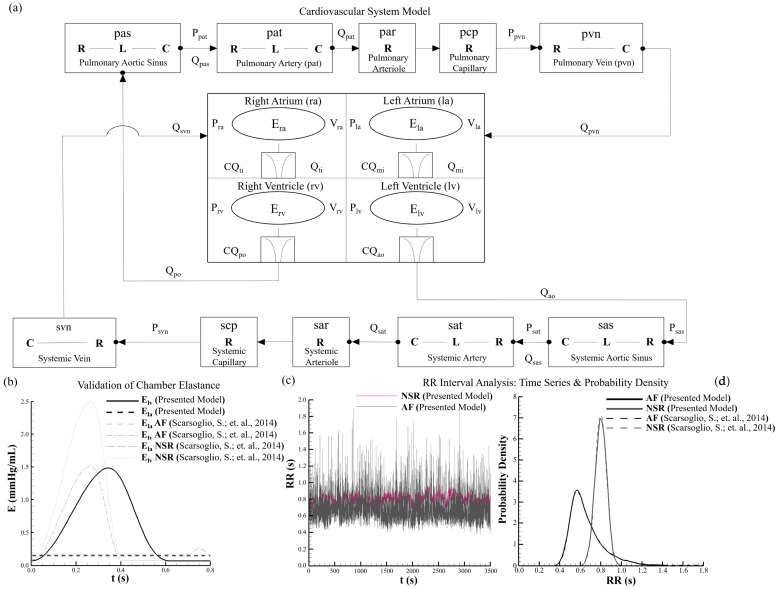
Lumped-parameter cardiovascular model and implementation verification. (**a**) Schematic of the closed-loop lumped-parameter cardiovascular model including the four cardiac chambers and the systemic and pulmonary circulations represented using resistance (R), compliance (C), and inertance (L) elements. Improved junction node labeling, clearer vascular connectivity, and directional flow arrows were incorporated to clarify the closed-loop circulation topology. (**b**) Implementation verification of the time-varying elastance functions for the left atrium and left ventricle through comparison with previously reported reference data [32], demonstrating physiologically consistent chamber mechanical behavior. (**c**) RR interval time-series representation for NSR and AF conditions showing regular ventricular activation under NSR and irregular beat-to-beat variability under AF. (**d**) Probability density distributions of RR intervals generated directly from the implemented stochastic RR interval generator using the model parameters described in the manuscript. The AF distribution demonstrated qualitative agreement with previously reported AF RR interval characteristics [32], thereby verifying correct implementation of the stochastic RR interval generation method.

The structure and implementation verification of the proposed model are summarized in Figure 1. Figure 1a illustrates the revised closed-loop lumped-parameter cardiovascular model with improved junction node labeling, clearer representation of pulmonary and systemic circulation topology, and directional arrows indicating the major blood flow pathways throughout the cardiovascular system. Figure 1b presents implementation verification of the time-varying elastance functions for the left atrium and left ventricle through comparison with previously reported reference data [32]. Figure 1c shows the RR interval time series for NSR and AF, highlighting the increased beat-to-beat variability under AF conditions. Figure 1d presents the corresponding probability density distributions of RR intervals, with NSR exhibiting a narrow distribution and AF showing a broader, asymmetric distribution, demonstrating the stochastic nature of the simulated AF rhythm. Additional mathematical derivations, parameter tables, and sensitivity analysis results are provided in the Supplementary Material.

### 2.1. Lumped-Parameter Cardiovascular Model

The cardiovascular system was represented by a closed-loop lumped-parameter model to describe the interactions among the cardiac chambers and the systemic and pulmonary circulations (Figure 1). In this approach, the hemodynamic behavior of the cardiovascular system was described using an electrical analog representation in which resistance (R), compliance (C), and inertance (L) elements represented vascular resistance, vessel elasticity, and blood inertial effects, respectively. The model consisted of four cardiac chambers, the right atrium, right ventricle, left atrium, and left ventricle, connected to the pulmonary and systemic vascular compartments. Flow variables represented blood flow between compartments, while pressure and volume were computed throughout the cardiac chambers and vascular compartments. Cardiac valves were represented as ideal diodes, allowing unidirectional blood flow between chambers and preventing regurgitant backflow during the cardiac cycle. The pulmonary circulation included the pulmonary artery, pulmonary arterioles, pulmonary capillaries, and pulmonary veins, whereas the systemic circulation consisted of the aortic sinus, systemic arteries, systemic capillaries, and systemic veins. This closed-loop configuration preserved total blood volume and enabled dynamic interaction between cardiac pumping behavior and vascular hemodynamics.

### 2.2. Time-Varying Elastance Model of Cardiac Chambers

The mechanical behavior of each cardiac chamber was described using the time-varying elastance formulation. In this framework, chamber pressure was determined from the instantaneous elastance and the difference between chamber volume and the unstressed volume. The atrial and ventricular elastance functions, denoted as E_la_, E_ra_, E_lv_, and E_rv_, varied periodically over the cardiac cycle to reproduce the phases of contraction and relaxation. Ventricular elastance increased rapidly during systole, followed by a gradual decrease during diastole, reflecting ventricular contraction and filling dynamics. In contrast, atrial elastance remained relatively low during most of the cardiac cycle with a modest increase during atrial contraction. Under AF conditions, the coordinated atrial contraction component was reduced within the elastance formulation to reproduce the diminished booster pump function characteristic of atrial fibrillation. To ensure correct implementation of the chamber mechanics, the elastance profiles generated by the present model were compared with previously reported reference data [32] (Figure 1b). The qualitative agreement between the simulated and reference elastance curves verified correct implementation of the time-varying elastance formulation and demonstrated physiologically consistent atrial and ventricular mechanical behavior.

### 2.3. Governing Equations

The hemodynamic behavior of the cardiovascular system was described using a lumped-parameter framework based on mass conservation and pressure–flow relationships. The pressure within each cardiac chamber was determined using the time-varying elastance formulation as given in Equation (1) [20]:

(1) $$P_{t}=P_{un}+E_{t}\left(V_{t}-V_{un}\right)$$

where (*P_t_*) denotes chamber pressure, (*E_t_*) is the time-varying elastance, (*V_t_*) is the instantaneous chamber volume, and (*V_un_*) represents the unstressed volume.

The conservation of mass governed the temporal evolution of ventricular volume shown in Equation (2) [20]:

(2) $$\frac{dV_{lv}}{dt}=Q_{mi}-Q_{ao}$$

where (*Q_mi_*) and (*Q_ao_*) represent the mitral inflow and aortic outflow, respectively.

Similar formulations based on mass conservation and time-varying elastance were applied to the left atrium, right atrium, and right ventricle, thereby ensuring consistent representation of all cardiac chambers within the closed-loop cardiovascular system. The complete set of governing equations, including atrial dynamics, right ventricular dynamics, valve flow relations, and vascular compartment equations, is provided in the Supplementary Sections S1 and S2.

Flow through the cardiac valves was defined using a pressure-driven relation with valve-dependent resistance and opening characteristics. The aortic valve flow was expressed (Equation (3)) [20] as follows:

(3) $$Q_{ao}=\left\{\begin{array}{ll} CQ_{ao}AR_{ao}\sqrt{P_{lv}-P_{sas}}, & if\;P_{lv}\geq P_{sas}\\ -CQ_{ao}AR_{ao}\sqrt{P_{sas}-P_{lv}}, & if\;P_{sas}<P_{lv} \end{array}\right.$$

The time-varying elastance of the left ventricle was defined in Equation (4) [20] as follows:

(4) $$E_{lv}\left(t\right)=E_{lv,min}+\frac{E_{lv,max}-E_{lv,min}}{2}e_{v}\left(t\right)$$

To reproduce the irregular ventricular rhythm characteristic of atrial fibrillation, RR intervals were generated using an exponentially modified Gaussian (EMG) distribution shown in Equation (5) [20]:

(5) $$p\left(RR;\mu_{G},\sigma_{G},\gamma \right)=\frac{\gamma }{2}e^{\mp \left(2\mu_{G}+\gamma \sigma_{G}^{2}-2RR\right)}\times erfc\left(\frac{\mu_{G}+\gamma \sigma_{G}^{2}-RR}{\sqrt{2}\sigma_{G}}\right)$$

Furthermore, left ventricular contractility was modulated based on the preceding RR intervals to capture beat-to-beat variability as given in Equation (6) [20]:

(6) $$E_{lv,max}=0.59\frac{RR1}{RR2}+0.91\;mmHg/mL$$

The complete set of governing equations, including atrial dynamics, right heart dynamics, valve flow relations, and vascular compartment equations, is provided in the Supplementary Material S1.1–S2.2. Additional hemodynamic quantities, including cardiac output, peak aortic flow, peak mitral inflow, and mean pulmonary venous pressure, were extracted from the simulated flow and pressure waveforms for quantitative comparison across remodeling conditions.

### 2.4. RR Interval Generation for Rhythm Simulation

Cardiac rhythm was defined by the RR interval sequence, which determined the timing of ventricular contractions. Two rhythm conditions were considered: NSR and AF. For the NSR condition, pink noise was used to generate physiological RR interval variability around a mean RR interval of 0.8 s (75 bpm), with a coefficient of variation of 0.07, thereby reproducing nearly periodic ventricular activation with minimal physiological variability. In contrast, AF was modeled using a stochastic RR interval sequence generated from an exponentially modified Gaussian (EMG) distribution with parameter values of μG = 0.5, σG = 0.5, and γ = 6 to reproduce the irregular ventricular response characteristic of atrial fibrillation. The generated RR intervals were analyzed using both time-series representation (Figure 1c) and probability density distributions (Figure 1d). The NSR sequence showed a narrow distribution centered on the mean RR interval, whereas the AF sequence exhibited a broader, asymmetric distribution reflecting increased beat-to-beat variability. The agreement between the simulated RR interval statistics and previously reported reference data [32] confirmed that the implemented stochastic generator successfully reproduced the temporal characteristics of atrial fibrillation. The corresponding EMG distribution parameters and RR interval statistics are summarized in Supplementary Table. These RR sequences were subsequently used as input to the cardiovascular model to investigate the hemodynamic differences between NSR and AF conditions. Furthermore, the stochastic RR interval sequences were simulated over multiple consecutive cardiac cycles to ensure adequate statistical representation of AF-induced hemodynamic variability.

### 2.5. Numerical Implementation

The governing nonlinear ordinary differential equations were solved using a variable-step, implicit solver (ode15s, MATLAB R2024a), which was well-suited to stiff cardiovascular systems. The time step was automatically adjusted to maintain numerical stability and solution accuracy. Simulations were performed over an extended sequence of cardiac cycles to capture the stochastic hemodynamic variability associated with atrial fibrillation adequately. A total of 30 consecutive AF cardiac cycles were simulated for each condition using stochastic RR interval sequences generated from the exponentially modified Gaussian distribution. The initial 10 transient cycles were excluded to eliminate initialization effects, and convergence toward statistical steady-state behavior was verified from the stabilization of pressure and volume responses across successive cycles. The reported hemodynamic results and pressure–volume relationships were obtained from the converged multi-cycle simulation ensemble rather than from a single cardiac cycle.

### 2.6. Model Verification and Physiological Consistency Assessment

The structure and implementation verification of the proposed cardiovascular model are summarized in Figure 1. Model implementation was assessed by comparing RR interval statistics, elastance behavior, and hemodynamic trends with previously reported physiological and computational studies [32]. The stochastic RR interval generator was first evaluated against established characteristics of cardiac rhythm variability. Under NSR conditions, RR intervals were reported to follow a Gaussian distribution with a coefficient of variation ranging from 0.05 to 0.13, indicating low physiological variability. In contrast, AF exhibited substantially higher variability, with a coefficient of variation of approximately 0.24, and RR intervals described using an exponentially modified Gaussian (EMG) distribution. As shown in Figure 1c, the simulated RR interval time series under NSR exhibited regular periodic behavior, whereas AF generated irregular beat-to-beat fluctuations. The corresponding probability density distributions in Figure 1d showed that NSR produced a narrow distribution centered on the mean RR interval. In contrast, AF produced a broader, asymmetric distribution with greater dispersion. These findings indicated that the implemented stochastic generator reproduced physiologically consistent AF-like temporal variability. The mechanical behavior of the cardiac chambers was further examined by comparing it with previously reported lumped-parameter formulations. Time-varying elastance functions have been widely used to represent cardiac chamber contraction and relaxation dynamics in reduced-order cardiovascular models. As shown in Figure 1b, the simulated elastance profiles for the left atrium and left ventricle demonstrated physiologically consistent trends: ventricular elastance increased during systole and decreased during diastole. Under AF conditions, atrial elastance remained reduced, reflecting diminished coordinated atrial contraction reported in previous studies. The qualitative agreement between the simulated and reference elastance curves verified the correct implementation of the adopted elastance formulation. The model hemodynamic outputs were also examined by comparing them with previously reported AF-related hemodynamic trends. Prior studies have shown that AF increases variability in pressure, flow, and cardiac output due to irregular ventricular filling and impaired atrial mechanical function. In the present simulations, AF produced pronounced beat-to-beat variability in ventricular pressure and flow, whereas NSR maintained stable periodic behavior. The distributions of cardiovascular variables became broader under AF conditions, indicating increased dispersion and variability. Similar trends have been reported in previous computational studies in which AF increased variability in cardiac output and ventricular ejection behavior. These findings demonstrated physiologically consistent hemodynamic trends associated with AF reported in previous studies. Finally, the overall model structure remained consistent with previously reported lumped-parameter cardiovascular frameworks in which the circulation was represented using resistance, compliance, and inertance elements coupled with time-varying elastance functions. The model preserved total blood volume and generated physiologically realistic pressure–volume relationships across all simulated conditions. The combined incorporation of stochastic RR interval variability and atrial mechanical impairment enabled the framework to reproduce key AF-related hemodynamic characteristics, including irregular ventricular response, reduced atrial contraction, and increased beat-to-beat hemodynamic variability.

## 3. Results and Discussion

### 3.1. Hemodynamic Comparison Between AF and NSR

**Figure 2:**
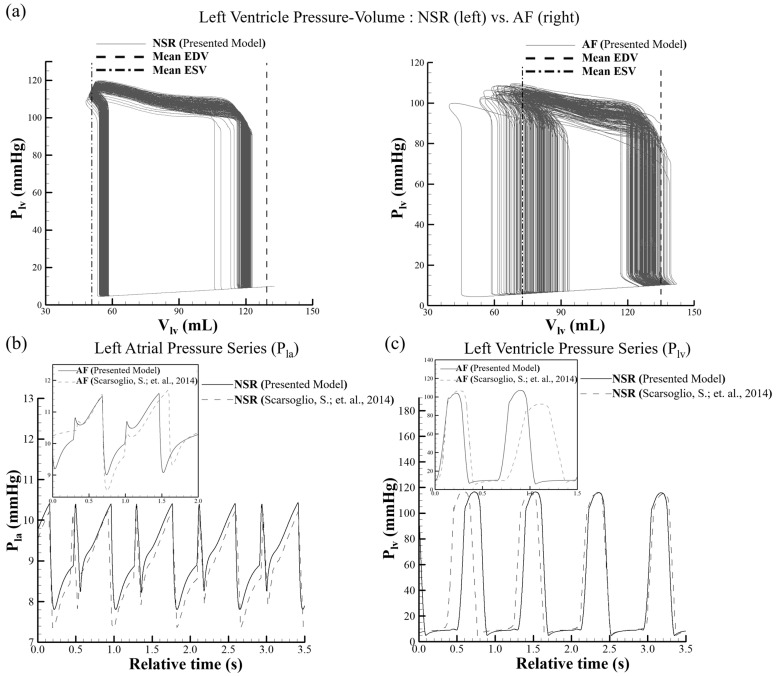
Left ventricular pressure–volume relationships and pressure dynamics under NSR and AF conditions. (**a**) Left ventricular pressure–volume (P–V) relationships demonstrating stable and repeatable ventricular behavior under NSR, whereas AF exhibited pronounced beat-to-beat variability in end-diastolic volume (EDV) and end-systolic volume (ESV) caused by stochastic RR interval variability and irregular ventricular filling dynamics. The AF pressure–volume relationships were obtained from overlaid multi-cycle simulations after excluding the initial transient cycles and after convergence toward statistical steady-state behavior. (**b**) Left atrial pressure (Pla) waveforms under NSR compared with reference data [32], showing periodic atrial mechanical activity associated with reservoir, conduit, and booster pump function; AF conditions (inset) demonstrated irregular pressure fluctuations with diminished coordinated atrial contraction. (**c**) Left ventricular pressure (P_lv_) waveforms under NSR compared with reference data [32], showing regular systolic and diastolic behavior; AF conditions (inset) demonstrated cycle-to-cycle variability in peak pressure and timing caused by irregular RR intervals and altered preload conditions.

The cardiac cycle was governed by the dynamic interaction between ventricular pressure and volume, which together determined the mechanical performance of the left ventricle under different rhythm conditions. To quantify the effects of AF on ventricular function, left ventricular pressure–volume (P–V) relationships, along with atrial and ventricular pressure time series, were analyzed and compared with normal sinus rhythm (NSR), as shown in Figure 2. In Figure 2a, the left ventricular P–V relationships demonstrated distinct differences between NSR and AF conditions. Under NSR, the P–V relationships exhibited a well-defined and consistent morphology with minimal beat-to-beat variation. The end-diastolic volume (EDV) and end-systolic volume (ESV) remained tightly clustered, indicating stable ventricular filling and ejection. The shape reflected efficient isovolumetric contraction and relaxation phases, with peak systolic pressure reaching approximately 120 mmHg. The presented AF pressure–volume relationships were obtained from overlaid multi-cycle simulations after statistical convergence, thereby allowing visualization of stochastic beat-to-beat hemodynamic variability induced by irregular RR intervals. The EDV varied over a wider range (approximately 60–130 mL), while ESV also showed increased spread, indicating inconsistent ventricular emptying. This behavior was primarily associated with stochastic beat-to-beat RR interval variability, which directly altered ventricular diastolic filling duration. Longer RR intervals increased ventricular filling time and produced larger end-diastolic volumes, whereas shorter RR intervals reduced filling duration and resulted in smaller ventricular volumes. Consequently, the AF pressure–volume relationships exhibited a broad distribution of EDV values, reflecting irregular ventricular filling dynamics characteristic of atrial fibrillation. The robustness of these remodeling-associated hemodynamic trends under different RR interval conditions was further evaluated through additional sensitivity analysis provided in the Supplementary Figure S1. This behavior was additionally influenced by the absence of coordinated atrial contraction, which impaired preload regulation and ventricular contractile consistency. Consequently, stroke volume fluctuated significantly during AF, suggesting reduced mechanical efficiency. Figure 2b presents the left atrial pressure (Pla) time series. Under NSR, the waveform exhibited periodic and well-defined features corresponding to the atrial reservoir, conduit, and booster pump phases. The pressure varied within a narrow physiological range of approximately 7.5–10.5 mmHg, with distinct peaks associated with atrial contraction. The simulated waveform demonstrated qualitative agreement with previously reported reference data [32] in both morphology and amplitude. Under AF conditions (inset), the atrial pressure waveform became irregular, with diminished peak definition and loss of periodicity. The absence of coordinated atrial contraction reduced the characteristic atrial “a-wave” and produced smoother but more erratic pressure fluctuations. This behavior was physiologically consistent with reduced atrial mechanical contribution to ventricular filling. Additional waveform-based physiological interpretation of left atrial mechanical behavior under NSR and AF conditions is provided in Supplementary Figure S2, including synchronized left atrial pressure, left atrial volume, signed transmitral flow, and valve timing relationships. Under NSR conditions, the waveform relationships demonstrated physiologically interpretable reservoir filling, passive conduit emptying, and late atrial booster pump contribution during ventricular filling. In contrast, AF conditions exhibited diminished atrial mechanical coordination, irregular transmitral flow timing, and increased beat-to-beat variability associated with stochastic RR interval dynamics. Figure 2c illustrates the left ventricular pressure (Plv) time series. Under NSR, ventricular pressure exhibited regular periodic cycles, with rapid systolic upstrokes and consistent peak pressures around 115–120 mmHg, followed by a sharp decline during diastole. The simulated waveform demonstrated qualitative agreement with the reference data [32], indicating physiologically consistent ventricular dynamics. Under AF conditions (inset), the pressure waveform showed clear cycle-to-cycle variability in both peak magnitude and timing. Systolic pressure peaks became reduced and irregular, whereas diastolic pressures exhibited variability due to inconsistent filling durations. These variations were directly associated with irregular RR intervals and altered preload conditions. Overall, the results demonstrated that AF disrupted ventricular mechanical stability. The combined effects of rhythm irregularity and loss of coordinated atrial contraction increased variability in ventricular volumes, reduced pressure consistency, and impaired pressure–volume relationship coherence. These findings highlighted the critical role of atrioventricular coupling in maintaining efficient cardiac function and demonstrated that AF-related hemodynamic alterations could be effectively represented using the present modeling framework.

### 3.2. Effect of Atrial Fibrillation Severity on Left Atrial and Ventricular Pressure–Volume Characteristics

**Figure 3:**
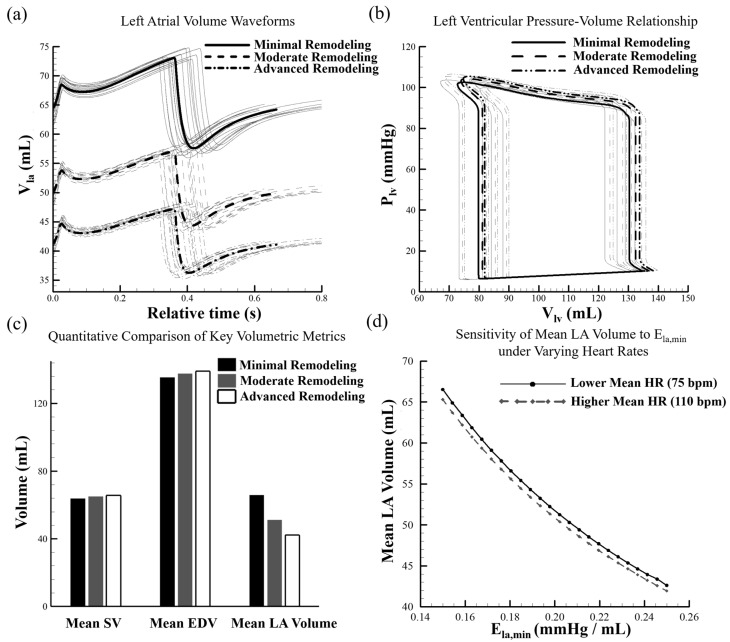
Hemodynamic response during AF with progressive atrial remodeling and sensitivity analysis. Atrial remodeling was characterized by progressive increases in minimum left atrial elastance (E_la,min_), representing increasing atrial stiffening and reduced compliance. A total of 30 AF cardiac cycles were simulated for each remodeling condition using stochastic RR interval sequences generated from the exponentially modified Gaussian distribution. The initial 10 transient cycles were excluded from analysis, and the remaining converged cycles were used for the multi-cycle visualizations. (**a**) Effective left atrial pressure–volume relationships demonstrating progressive leftward and upward shifts with increasing remodeling severity, indicating reduced atrial compliance and increased chamber stiffness. (**b**) Representative converged left ventricular P–V relationships from selected AF cycles showing preserved systolic pressure together with progressive increases in end-diastolic volume, reflecting compensatory ventricular adaptation through the Frank–Starling mechanism despite reduced atrial mechanical contribution. (**c**) Multi-cycle left atrial volume waveforms illustrating beat-to-beat variability and progressive attenuation of volume oscillation amplitude with increasing remodeling severity, indicating impaired atrial reservoir and contractile function. (**d**) Quantitative comparison of key volumetric parameters demonstrating preserved stroke volume, increased ventricular end-diastolic volume, and progressive reduction in mean left atrial volume under increasing atrial remodeling severity. Additional sensitivity analysis results under different heart-rate conditions are provided in Supplementary Figure S1.

AF was associated with disruption of coordinated electrical activation and mechanical contraction of the atria, leading to altered atrioventricular coupling and impaired ventricular filling dynamics. Under physiological conditions, ventricular filling was regulated through three sequential atrial functional phases: reservoir filling during ventricular systole, passive conduit flow during early diastole, and active atrial contraction during late diastole. These phases maintained ventricular preload and stable cardiac output by enabling efficient blood transfer into the ventricle. With progression of AF-associated structural remodeling, atrial electrical activity progressively lost coordinated activation, mechanical contraction was reduced, and booster pump function became diminished. Consequently, ventricular filling efficiency was impaired, and atrioventricular mechanical interaction became altered. Under AF conditions, coordinated atrial contraction became diminished, resulting in attenuation of active atrial mechanical contribution to ventricular filling. Consequently, the simulated left atrial pressure–volume behavior became less dynamic and increasingly dominated by passive reservoir and conduit characteristics. This behavior was physiologically consistent with impaired atrial mechanical activity and weakened atrioventricular coupling commonly observed during AF. To quantify these effects, pressure–volume relationships, atrial volume dynamics, and volumetric parameters were evaluated under minimal, moderate, and advanced remodeling conditions. For each remodeling condition, a total of 30 AF cardiac cycles were simulated using stochastic RR interval sequences generated from the exponentially modified Gaussian distribution. The initial 10 transient cycles were excluded from analysis, and the remaining converged cycles were used for the multi-cycle evaluations. The left atrial pressure–volume relationships in Figure 3a demonstrated progressive alterations in atrial mechanical behavior with increasing remodeling severity. Under minimal remodeling conditions, atrial pressure ranged from 8.8 to 11.6 mmHg, while atrial volume ranged from 58 to 73 mL, indicating preserved compliance and effective chamber expansion. As remodeling progressed, the pressure–volume relationship shifted toward higher pressures and lower operating volumes. Under moderate remodeling conditions, atrial pressure increased to 11.9 mmHg with a reduced volume range of 45–58 mL. In the advanced remodeling condition, pressure further increased to 12.1 mmHg while volume decreased to 35–48 mL. This leftward shift and increased slope of the pressure–volume relationship indicated reduced atrial compliance and increased chamber stiffness. These changes were physiologically consistent with atrial remodeling characterized by increased fibrosis and reduced chamber compliance, in which higher pressure was required to accommodate smaller chamber volumes. The left ventricular pressure–volume relationship shown in Figure 3b demonstrated that ventricular systolic function remained largely preserved across all remodeling stages. To preserve visualization clarity, only representative converged AF cycles were displayed for the ventricular pressure–volume relationships. Peak ventricular pressure remained within 100–105 mmHg, indicating sustained ventricular contractile capability. However, progressive changes were observed during diastolic filling. End-diastolic volume increased from 130 mL under minimal remodeling to 132 mL and 134 mL under moderate and advanced remodeling conditions, respectively. This increase indicated a compensatory ventricular filling response that partially offset the reduced atrial contribution to ventricular preload. As coordinated atrial contraction became diminished, ventricular filling became increasingly dependent on passive pressure gradients during early diastole. Consequently, ventricular preload increased slightly to preserve stroke volume through the Frank–Starling mechanism. Increased end-diastolic volume enhanced myocardial fiber stretch and supported maintenance of ventricular output despite impaired atrial mechanical function. The temporal variation of left atrial volume is illustrated in Figure 3c. Under minimal remodeling conditions, atrial volume ranged from 58 to 74 mL, producing a distinct oscillatory waveform corresponding to the reservoir, conduit, and contraction phases. As remodeling severity increased, the amplitude of this waveform decreased progressively. Under moderate remodeling conditions, volume ranged from 45 to 57 mL, whereas in the advanced condition, it decreased further to 36–47 mL. This attenuation indicated progressive reduction in atrial reservoir capacity and loss of active atrial contraction, with the atrium increasingly functioning as a passive conduit chamber. A quantitative comparison of volumetric parameters is presented in Figure 3d. Mean stroke volume remained relatively stable, increasing slightly from 64 mL under minimal remodeling to 66 mL under advanced remodeling, indicating preservation of global cardiac output despite progressive atrial dysfunction. Mean end-diastolic volume increased from 130 mL to 134 mL, further supporting compensatory ventricular preload adaptation through the Frank–Starling mechanism. In contrast, mean left atrial volume decreased from 66 mL to 42 mL, corresponding to a reduction of 36.4%, indicating substantial loss of atrial reservoir function. These findings suggested redistribution of blood volume from the atrium to the ventricle during progressive remodeling. Overall, progressive atrial remodeling was associated with a consistent hemodynamic pattern characterized by reduced atrial compliance, diminished volume oscillation, and increased ventricular end-diastolic volume. Although ventricular systolic pressure generation remained preserved, impaired atrial mechanics resulted in altered preload regulation and weakened atrioventricular coupling. The loss of atrial booster pump function increased reliance on passive ventricular filling, necessitating compensatory ventricular adaptation to maintain stroke volume. These results highlighted the critical role of atrial mechanical function in maintaining efficient cardiac performance and provided mechanistic insight into hemodynamic alterations associated with AF progression. To provide a more comprehensive characterization of the simulated AF hemodynamic response, additional global cardiovascular and flow-related quantities were evaluated under the different remodeling conditions. Specifically, cardiac output (CO), peak aortic flow (Q_ao,peak_), peak mitral inflow (Q_mi,peak_), and mean pulmonary venous pressure (Ppvn) were extracted from the model outputs. These quantities were selected to assess systemic pumping performance, ventricular ejection behavior, atrioventricular filling dynamics, and upstream pulmonary venous loading, respectively. The results demonstrated mild but consistent increases in all four quantities with progressive atrial remodeling. Cardiac output increased from 5.47 L/min under minimal remodeling to 5.62 L/min under advanced remodeling, while peak aortic flow increased from 1106.9 mL/s to 1121.2 mL/s. Similarly, peak mitral inflow increased from 876.5 mL/s to 889.9 mL/s, and mean pulmonary venous pressure increased from 10.71 mmHg to 11.00 mmHg. Although the absolute magnitude of these changes remained relatively small, the observed trends remained consistent across all remodeling conditions, indicating that progressive atrial stiffening exerted measurable effects on ventricular filling and global circulatory dynamics. To further evaluate the robustness of the observed remodeling-associated trends, an additional sensitivity analysis was performed by varying E_la,min_ continuously over the investigated physiological range under both normal-rate (75 bpm) and elevated-rate (110 bpm) AF conditions. The results demonstrated that the principal hemodynamic trends, including reduced atrial compliance, attenuation of atrial volume oscillation, and compensatory increases in ventricular end-diastolic volume, remained qualitatively consistent across the investigated parameter range. The corresponding sensitivity analysis results are presented in Supplementary Figure S1. Additional local sensitivity analyses involving perturbations of the mitral valve flow coefficient, pulmonary venous resistance, and grouped systemic vascular resistance are provided in Supplementary Figures S3–S5. Unlike previous studies that primarily focused on rhythm-induced variability, the present framework enables systematic evaluation of the combined effects of stochastic RR-interval variability and progressive atrial structural remodeling. This integrated approach provides additional insight into the interplay between atrial mechanical impairment and ventricular compensation, which remains difficult to isolate directly in experimental or clinical settings.

### 3.3. Left Atrial Volume Dynamics and Pressure–Volume Behavior Under Progressive Remodeling Conditions

**Figure 4:**
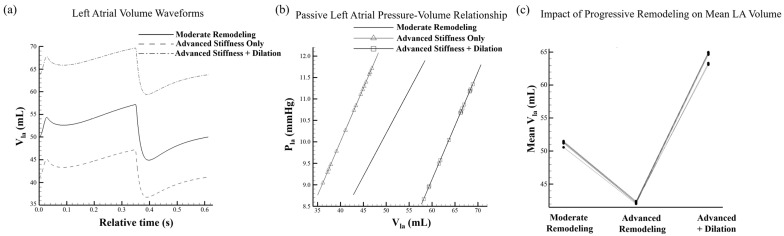
Left atrial volume dynamics and pressure–volume behavior under progressive remodeling conditions. (**a**) Temporal variation of left atrial (LA) volume over the cardiac cycle for moderate remodeling, advanced stiffness-only remodeling, and advanced stiffness with dilation conditions. Moderate remodeling demonstrated peak and minimum LA volumes of 57 mL and 45 mL, respectively, whereas advanced stiffness reduced these volumes to 47 mL and 37 mL, indicating reduced atrial compliance and reservoir function. In contrast, advanced stiffness with dilation shifted the waveform toward higher operating volumes (70 mL peak and 60 mL minimum), without restoring effective dynamic volume variation. (**b**) Effective left atrial pressure–volume relationships illustrating progressive increases in effective atrial elastance with remodeling severity. The P–V relationship slope increased from 0.23 mmHg/mL under moderate remodeling to 0.30 mmHg/mL under advanced stiffness conditions, indicating increased myocardial stiffness and reduced compliance. Under dilation conditions, the P–V relationship shifted toward higher operating volumes while maintaining elevated elastance, demonstrating that geometric enlargement did not reduce intrinsic atrial stiffness. (**c**) Quantitative comparison of mean LA volume across remodeling conditions showing progressive reduction with increasing stiffness and increased chamber enlargement under dilation conditions, indicating that atrial stiffening and geometric remodeling independently influenced atrial mechanical behavior and volumetric response.

The LA acted as a dynamic chamber that regulated ventricular filling through its reservoir, conduit, and booster pump functions, with its mechanical behavior governed by atrial compliance, myocardial stiffness, and structural adaptation. Under pathological conditions, these functional characteristics became altered, as reflected in the LA volume waveforms, effective pressure–volume relationship analyses, and quantitative comparisons presented in Figure 4. In the moderate remodeling condition, the LA volume waveform exhibited a progressive rise during the reservoir phase, reaching a peak of 57 mL at a relative time of 0.32 s, followed by a decline to a minimum of 45 mL during atrial emptying, resulting in a total LA stroke volume of 12 mL (Figure 4a). In the advanced stiffness-only condition, peak and minimum LA volumes decreased to 47 mL and 37 mL, respectively, with a corresponding stroke volume of 10 mL, representing a reduction relative to the moderate condition. This reduction in both peak and minimum volumes indicated diminished atrial compliance associated with progressive myocardial stiffening. In the advanced stiffness with dilation condition, the waveform shifted toward higher volumes, with peak and minimum values of 70 mL and 60 mL, respectively. Despite this geometric enlargement, LA stroke volume remained limited to 10 mL, indicating that increased chamber size did not restore effective reservoir or conduit function. The pressure–volume relationship analysis provided additional insight into atrial mechanical behavior across the three remodeling conditions (Figure 4b). Under moderate remodeling conditions, a near-linear pressure–volume relationship was observed over a volume range of 45–58 mL and a pressure range of 9.0–12.0 mmHg, corresponding to an effective elastance of 0.23 mmHg/mL. With progressive stiffening, the pressure–volume relationship slope increased to 0.30 mmHg/mL and shifted toward lower operating volumes (37–47 mL), reflecting restricted atrial expansion and reduced compliance. In the dilation condition, the pressure–volume relationship shifted toward higher operating volumes (60–70 mL), whereas elastance remained elevated at 0.29 mmHg/mL. This behavior indicated that geometric enlargement did not reduce intrinsic myocardial stiffness and that elevated elastance persisted despite structural adaptation. Quantitative comparison of mean LA volume demonstrated a decrease from 51 mL in the moderate remodeling condition to 42 mL in the advanced stiffness-only condition, indicating reduced atrial filling capacity. In contrast, the advanced stiffness with dilation condition exhibited an increased mean LA volume of 64 mL, reflecting chamber enlargement without corresponding improvement in functional output (Figure 4c). These findings demonstrated that myocardial stiffening reduced both the amplitude and operating range of LA volume oscillation, whereas dilation increased absolute chamber size without enhancing dynamic atrial mechanical performance. Overall, the findings indicated that structural stiffening and geometric enlargement contributed independently to atrial dysfunction. While progressive stiffening reduced atrial compliance and impaired volume variation, chamber dilation increased atrial size without restoring effective mechanical performance. These observations highlighted the distinct and complementary roles of mechanical and structural remodeling in determining atrial function under pathological conditions.

## 4. Limitations

Several limitations were associated with the present study. First, the cardiovascular system was represented using a lumped-parameter framework in which spatial variations in flow and pressure were not resolved. Consequently, localized hemodynamic phenomena and complex three-dimensional flow patterns were not captured. In addition, the left atrium was represented using a global time-varying elastance formulation. Accordingly, the simulated left atrial pressure–volume relationships should be interpreted as effective global chamber behavior rather than fully resolved physiological atrial pressure–volume loop morphology. Accordingly, the present framework was intended primarily as a mechanistic in silico model to investigate global hemodynamic trends and atrioventricular coupling under controlled AF conditions, rather than detailed patient-specific atrial mechanics. Second, cardiac valves were modeled as ideal diodes; therefore, valve dynamics such as regurgitation, leaflet inertia, and opening–closing delays were not considered. Third, AF was represented using stochastic RR interval sequences and modified atrial elastance, whereas detailed electrophysiological mechanisms governing arrhythmia initiation and propagation were not explicitly incorporated. Fourth, the model was not calibrated using patient-specific clinical measurements and therefore was not intended as a patient-specific predictive framework. Although inter-patient variability and differences in AF severity may influence the absolute magnitudes of simulated quantities, the proposed model consistently reproduced the principal relationships among rhythm irregularity, atrial remodeling, ventricular filling dynamics, and compensatory ventricular adaptation observed throughout the investigated conditions. Although expanded local sensitivity analyses were performed for multiple physiologically relevant parameters, including minimum left atrial elastance (E_la,min_), mitral valve flow coefficient (CQ_mi_), pulmonary venous resistance (R_pvn_), and grouped systemic vascular resistance (SVR), the present analysis remained limited to isolated local parameter perturbations. A comprehensive global multi-parameter sensitivity analysis and uncertainty quantification involving ventricular elastance, vascular compliance, valve properties, pulmonary circulation parameters, and RR variability characteristics would still be required to characterize broader uncertainty and assess robustness. Finally, structural remodeling was primarily reflected in changes in atrial elastance and therefore did not explicitly account for heterogeneous fibrosis distribution, anisotropic myocardial properties, or regional atrial tissue heterogeneity. Despite these limitations, the proposed framework captured key global hemodynamic trends and provided mechanistic insight into the interaction between atrial dysfunction and ventricular adaptation during AF. Future work should incorporate subject-specific parameter estimation, more detailed valve and electrophysiological modeling, and multiscale approaches to improve further physiological fidelity, individualized prediction capability, and translational applicability.

## 5. Conclusions

This study demonstrated that AF altered cardiac hemodynamics through the combined effects of rhythm irregularity and progressive atrial structural remodeling. The loss of coordinated atrial contraction and irregular RR intervals introduced significant beat-to-beat variability in ventricular preload, systolic pressure, and stroke volume, thereby reducing mechanical efficiency. In parallel, progressive atrial stiffening reduced atrial compliance and reservoir function, as evidenced by a 36.4% reduction in left atrial volume and a leftward shift in pressure–volume relationships. Ventricular stroke volume remained within a narrow range (64–66 mL) through compensatory increases in end-diastolic volume (130–134 mL), indicating redistribution of blood volume toward the ventricle. The proposed closed-relationships cardiovascular framework enabled direct quantification of the coupled effects of rhythm irregularity and atrial mechanical impairment on atrioventricular coupling, ventricular filling dynamics, and compensatory ventricular adaptation. Additional flow-related metrics further demonstrated that progressive atrial stiffening produced mild but consistent alterations in global cardiovascular dynamics and ventricular filling behavior. Overall, these findings provide mechanistic insight into AF-related hemodynamic dysfunction and highlight the critical role of atrial mechanics in maintaining efficient cardiac performance. Future work will focus on improving the physiological fidelity and clinical applicability of the proposed model through incorporation of patient-specific parameter estimation, advanced valve dynamics, and multiscale atrial remodeling representations. In addition, the framework may be extended toward biosensor-assisted cardiovascular monitoring and diagnostic applications through integration of simulated hemodynamic variables with measurable physiological signals.

## Data Availability

The original contributions presented in this study are included in the article/Supplementary Material. Further inquiries can be directed at the corresponding authors.

## Supplementary Materials

The following supporting information can be downloaded at: https://www.mdpi.com/article/10.3390/bioengineering13060639/s1. References [38,39] are cited in the supplementary materials.

## Nomenclature

**Symbol**	**Description**
A	sectional area
C	compliance
CQ	flow coefficient
DT	Time step
E	elastance
I	inertial moment of rotating
K,k	coefficient
L	inertance
M	mass
P	pressure
Q	flow rate
R	resistance
T	time; heart period
**Subscripts**	**Description**
0	initial value; offset value; value for unstressed condition
ao	aortic valve
bm	velocity effect on the valve dynamics, due to blood motion
d	diastolic phase
e	elastance action
ea	elastance of atrium
ev	elastance of ventricle
f	frictional action
fr	frictional effect on the valve dynamics, due to resistance of neighboring tissue
la	left atrium
lv	left ventricle
max	maximum value
min	minimum value
mi	mitral valve
p	effect of pressure force
par	pulmonary arterioles
pas	pulmonary aortic sinus
pat	pulmonary artery
pav	right annulus fibrosus
pcp	pulmonary capillary
po	pulmonary aortic valve
pr	pressure effect on the valve dynamics
pvc	pulmonary venae cavae
pvn	pulmonary vein
pwb	beginning of P wave
pww	duration of P wave
ra	right atrium
rv	right ventricle
s	systolic phase
s1	peak of systolic phase
s2	end of systolic phase
sar	systemic arterioles
sas	systemic aortic sinus
sat	systemic artery
sav	left annulus fibrosus
scp	systemic capillary
ss	effect of shear stress on the valve dynamics
st	strain action
svc	systemic venae cavae
svn	systemic vein
ti	tricuspid valve
vo	vortex effect on the valve dynamics
