# Supplementary material for: A Lumped-Parameter Cardiovascular Model for Investigating Hemodynamic Alterations During Atrial Fibrillation
Source: Bioengineering (Basel). 2026 May 29;13(6):639. doi: 10.3390/bioengineering13060639 (PMC13295863; doi:10.3390/bioengineering13060639)
Supplement: Supplementary file 1 [file bioengineering-13-00639-s001.zip › bioengineering-4278228-supplementary.pdf]

bioengineering

MDPI

Supplementary Material

A Lumped-Parameter Cardiovascular Model for Investigating Hemodynamic Alterations During Atrial Fibrillation

Prashant Kishor Sharma †, Yu-Chien Tung †, and Chia-Yuan Chen *

Department of Mechanical Engineering, National Cheng Kung University, Tainan 701, Taiwan; prashant94580@gmail.com (P.K.S.); n16141010@gs.ncku.edu.tw (Y.-C.T.)

† These authors contributed equally to this work.

* Correspondence: chiayuac@mail.ncku.edu.tw

Supplementary File Contains:

Supplementary Figures

Mathematical Model Overview

Parameters Table

Supplementary Figures

Additional local sensitivity analyses were performed to evaluate the robustness of the simulated hemodynamic response to perturbations in multiple physiologically relevant model parameters. Sensitivity analyses were conducted for minimum left atrial elastance (Ela,min), mitral valve flow coefficient (CQ_{mi}), pulmonary venous resistance (R_{pvn}), and grouped systemic vascular resistance (SVR) at two AF heart rates (75 bpm and 110 bpm). During each analysis, only the target parameter was varied, while the remaining model parameters were held constant.

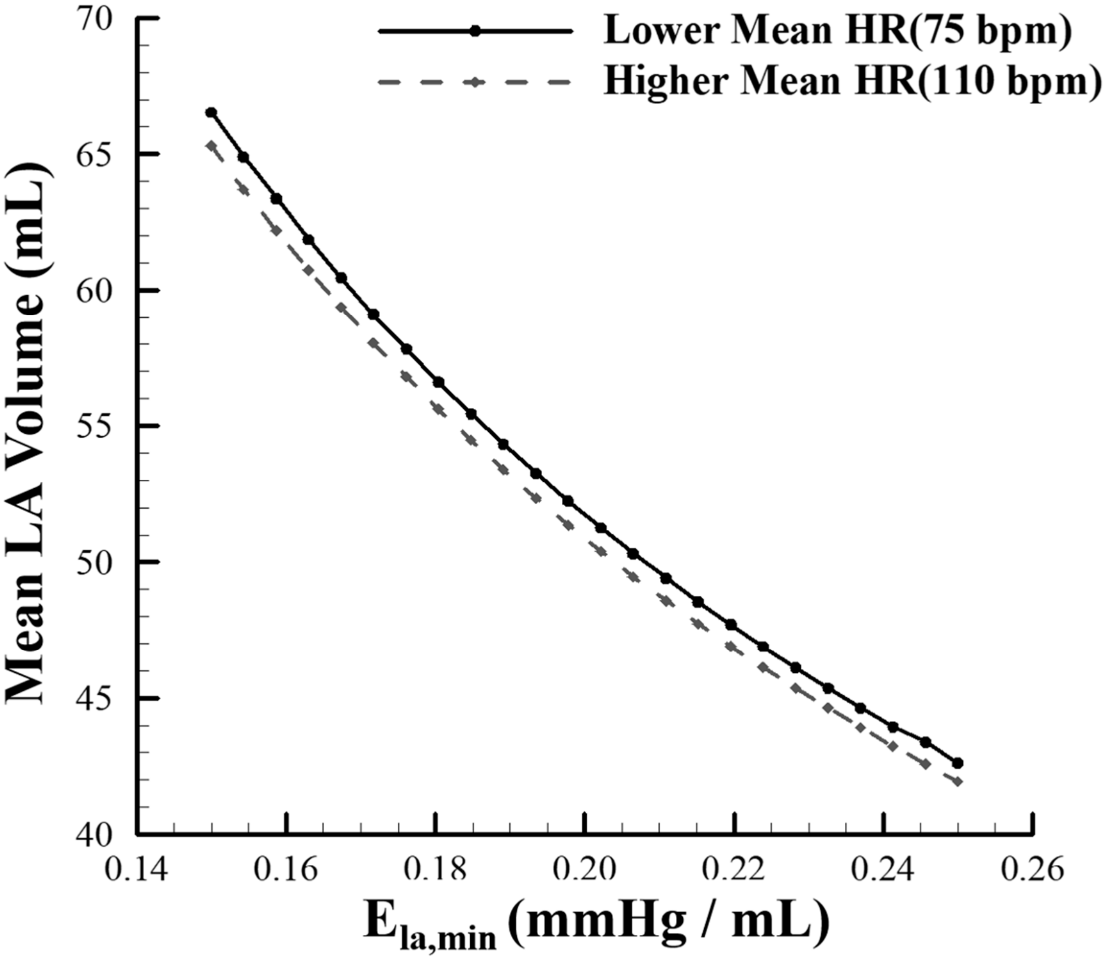

Supplementary Figure S1. Sensitivity analysis of mean left atrial (LA) volume under progressive atrial stiffening conditions. *The minimum left atrial elastance parameter (Ela,min) was varied continuously from 0.15 to 0.25 mmHg/mL to evaluate the influence of increasing atrial stiffness on LA volume behavior. Two mean heart-rate conditions were investigated: lower mean heart-rate AF (75 bpm) and higher mean heart-rate AF (110 bpm). Progressive increases in Ela, min produced a consistent reduction in mean LA volume under both heart-rate conditions, indicating reduced atrial compliance and impaired reservoir function with increasing remodeling severity. Although absolute LA volume remained lower under elevated heart-rate conditions, the overall remodeling-associated trends remained qualitatively consistent across the investigated parameter range.*

https://doi.org/10.3390/xxxxx

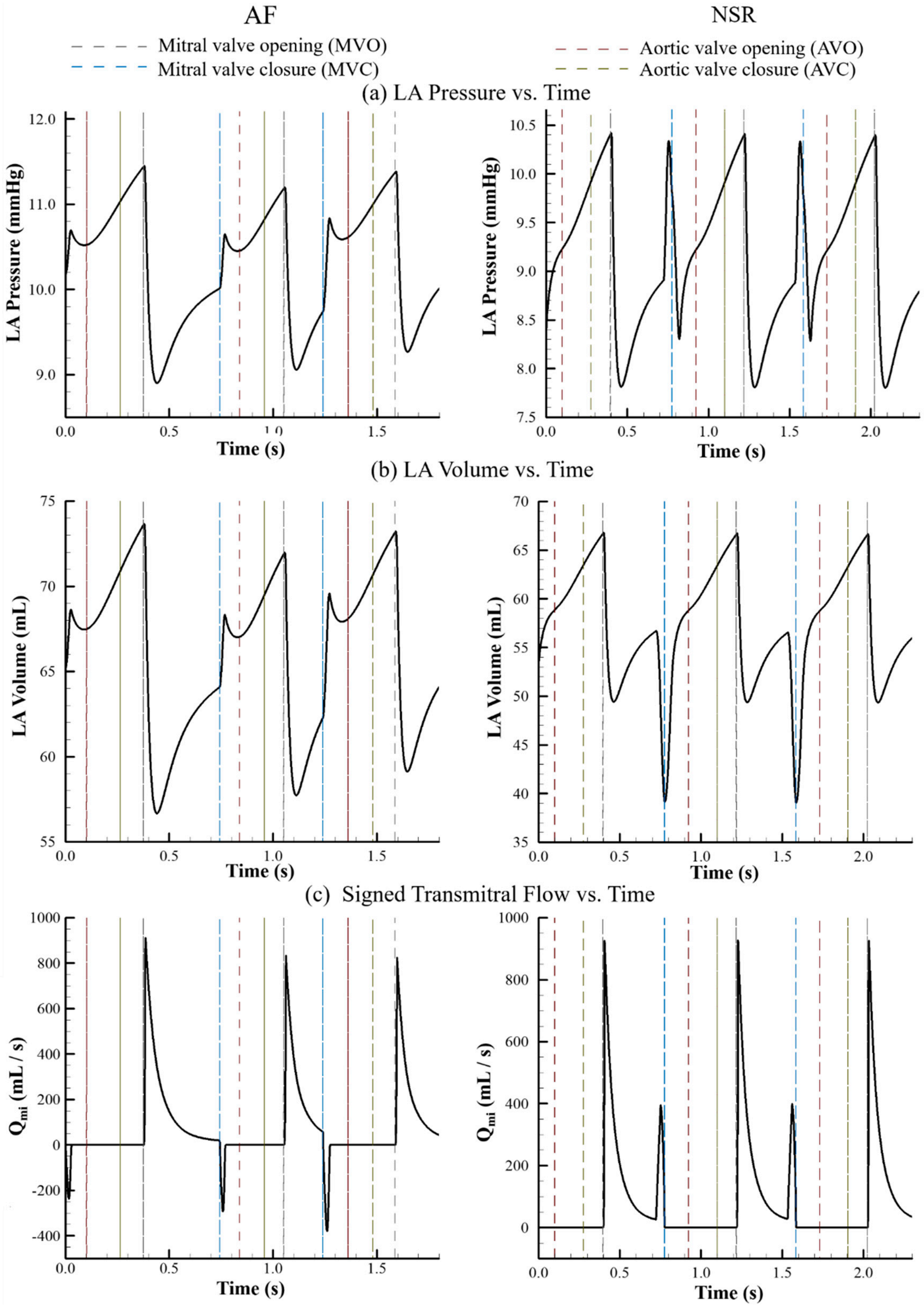

Supplementary Figure S2. Time-domain waveform analysis of left atrial dynamics under atrial fibrillation (AF) and normal sinus rhythm (NSR) conditions. *The left column presents the AF simulation results, whereas the right column presents the NSR simulation results. The three rows show (a) left atrial (LA) pressure, (b) LA volume, and (c) signed transmitral flow (Q_{mi}), respectively. Vertical dashed lines indicate mitral valve opening (MVO), mitral valve closure (MVC), aortic valve opening (AVO), and aortic valve closure (AVC) events identified from the simulated valve dynamics and flow signals. Under NSR conditions, the waveforms demonstrated periodic and coordinated atrial filling and emptying behavior with regular transmitral flow timing. In contrast, AF conditions exhibited increased beat-to-beat variability, altered atrial filling*

dynamics, and irregular transmitral flow behavior associated with stochastic RR interval variation and impaired atrial mechanical coordination. The supplementary waveform analysis was provided to support physiological interpretation of left atrial mechanical behavior and valve timing relationships in the lumped-parameter cardiovascular model.

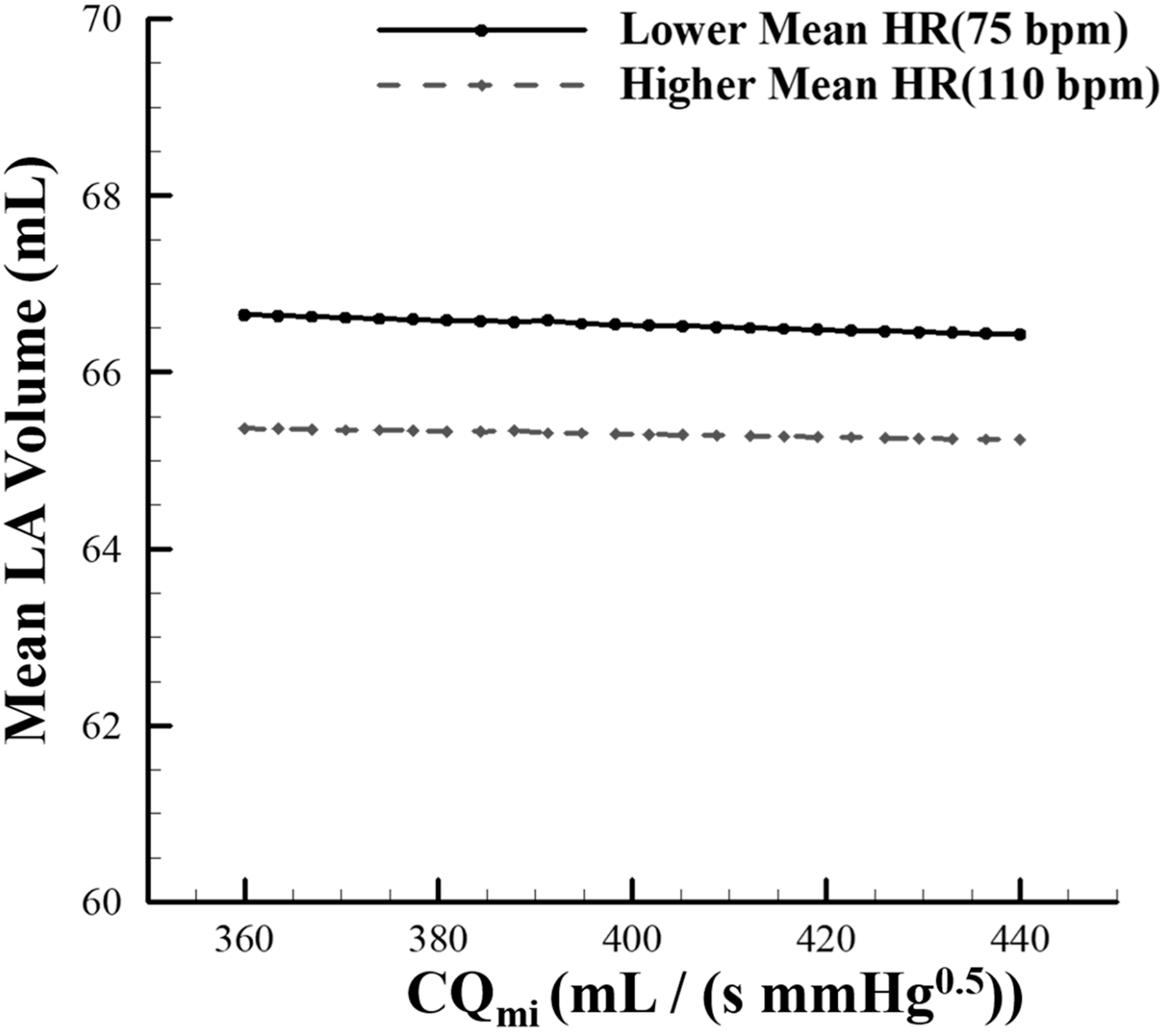

Supplementary Figure S3. Sensitivity analysis of mean left atrial (LA) volume to variation in mitral valve flow coefficient (CQ_{mi}). *The mitral valve flow coefficient parameter (CQ_{mi}) was varied over the range of 360–440 mL/(s·mmHg$^{0.5}$), corresponding to ±10% around the baseline value, to evaluate the influence of mitral valve flow characteristics on LA volume behavior. Two mean heart-rate AF conditions were investigated: lower mean heart-rate AF (75 bpm) and higher mean heart-rate AF (110 bpm). The results demonstrated minimal variation in mean LA volume across the investigated CQ_{mi} range, indicating relatively low sensitivity of the simulated atrial volume response to moderate perturbations in mitral valve flow coefficient.*

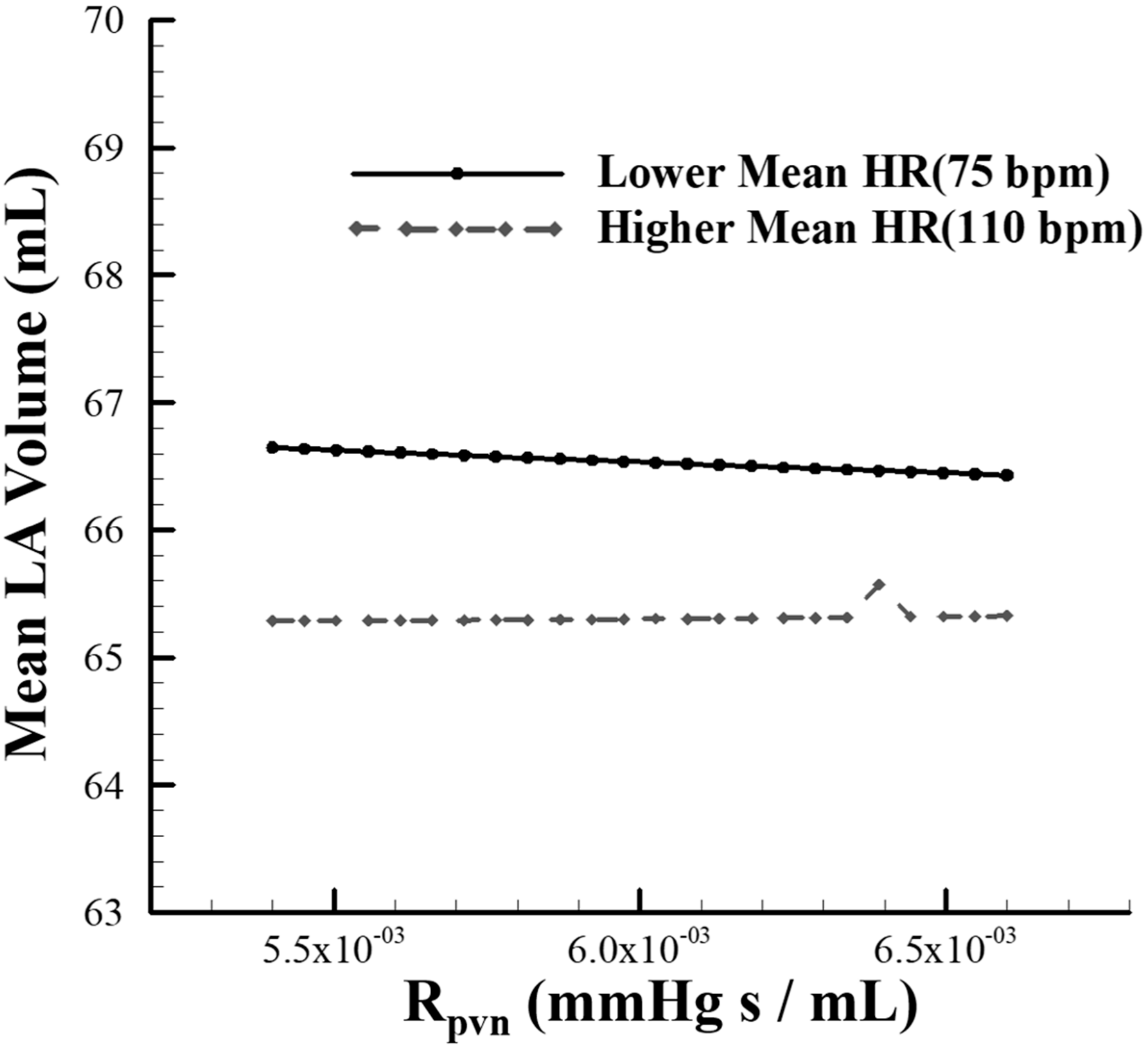

Supplementary Figure S4. Sensitivity analysis of mean left atrial (LA) volume to variation in pulmonary venous resistance (R_{pvn}). *The pulmonary venous resistance parameter (R_{pvn}) was individually perturbed by ±10% around its baseline value to evaluate the influence of pulmonary venous loading conditions on LA volume behavior. Two mean heart-rate AF conditions were investigated: lower mean heart-rate AF (75 bpm) and higher mean heart-rate AF (110 bpm). The results demonstrated only minor variation in mean LA volume across the investigated R_{pvn} range, indicating limited sensitivity of the simulated atrial volume response to moderate pulmonary venous resistance perturbations.*

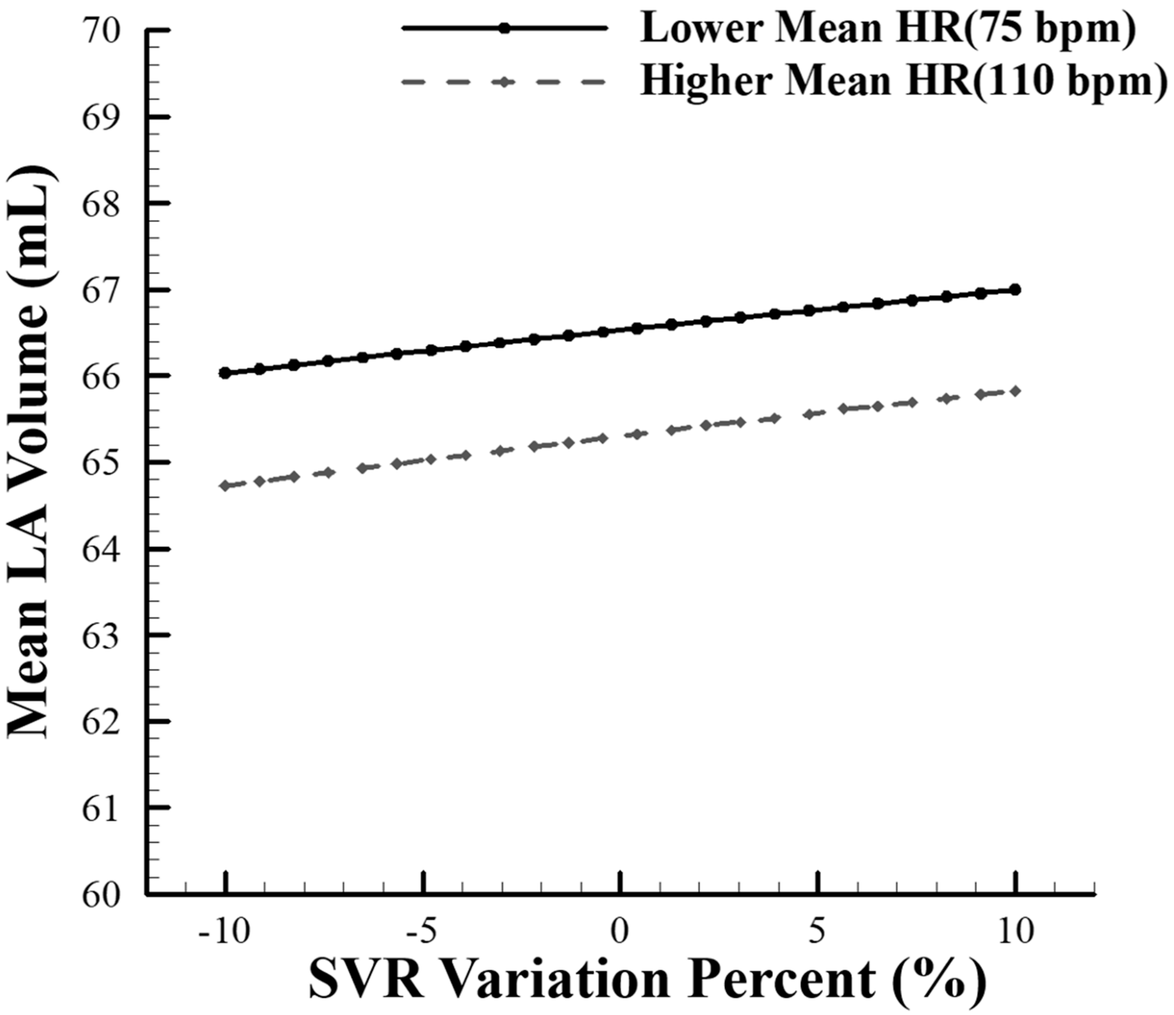

Supplementary Figure S5. Sensitivity analysis of mean left atrial (LA) volume under grouped systemic vascular resistance (SVR) variation conditions. *To evaluate the influence of systemic vascular loading on atrial volume behavior, the major systemic vascular resistance parameters were synchronously perturbed using a group-scaled factor within ±10% of baseline, while preserving the relative resistance distribution among the systemic compartments. Two mean heart-rate AF conditions were investigated: lower mean heart-rate AF (75 bpm) and higher mean heart-rate AF (110 bpm). Progressive increases in systemic vascular resistance produced mild increases in mean LA volume under both heart-rate conditions, indicating modest sensitivity of atrial volume to systemic vascular loading.*

S1. Cardiac Chamber Modeling

S1.1. Heartbeat Interval Model

The irregular heartbeat dynamics associated with AF were represented using an EMG distribution. This formulation allowed the reproduction of stochastic RR interval variability observed in AF conditions. The probability density function of the RR interval was defined [S1] as:

$$p(RR;\mu_G,\sigma_G,\gamma)=\frac{\gamma}{2}e^{\mp(2\mu_G+\gamma\sigma_G^2-2RR)}\times erfc\left(\frac{\mu_G+\gamma\sigma_G^2-RR}{\sqrt{2}\sigma_G}\right) \tag{S1}$$

where μ_G, σ_G, and γ represent the mean, standard deviation, and exponential rate parameter, respectively.

S1.2. Left Ventricular Contractility Model

To account for beat-to-beat variability in ventricular function during AF, the maximum elastance of the left ventricle was defined as a function of the two preceding RR intervals. This relationship enabled incorporation of memory effects in myocardial contractility [S2]:

$$E_{lv,max}=0.59\frac{RR1}{RR2}+0.91\ mmHg/ml \tag{S2}$$

where $RR1$ and $RR2$ denote the immediately preceding cardiac intervals.

S1.3. Left Atrium

The left atrium was modeled using conservation of mass and a time-varying elastance formulation. The governing equations [S3] describing volume variation and pressure–volume relationship were given as:

$$\frac{dV_{la}}{dt}=Q_{pvn}-Q_{mi} \tag{S3}$$

$$P_{la}=P_{la,un}+E_{la}\left(V_{la}-V_{la,un}\right) \tag{S4}$$

The mitral valve flow was defined as:

$$Q_{mi}=\begin{cases}CQ_{mi}AR_{mi}\sqrt{P_{la}-P_{lv}}, & if\ P_{la}\geq P_{lv}\\ -CQ_{mi}AR_{mi}\sqrt{P_{lv}-P_{la}}, & if\ P_{la}<P_{lv}\end{cases} \tag{S5}$$

https://doi.org/10.3390/xxxxx

The time-varying elastance of the left atrium was expressed as:

$$E_{la}(t) = E_{la,min} + \frac{E_{la,max} - E_{la,min}}{2} e_a(t) \tag{S6}$$

$$e_a(t) = \begin{cases} 0, & if\ 0 \le t \le T_{ac} \\ 1 - cos\left(\frac{t - T_{ac}}{RR - T_{ac}} 2\pi\right) & if\ T_{ac} < RR \end{cases} \tag{S7}$$

The mitral valve opening area ratio was defined as:

$$AR_{mi} = \frac{(1 - cos\ \theta_{mi})^2}{(1 - cos\ \theta_{max})^2} \tag{S8}$$

S1.4. Left Ventricle

The left ventricle was described using a time-varying elastance model coupled with valve flow dynamics [S3]:

$$\frac{dV_{lv}}{dt} = Q_{mi} - Q_{ao} \tag{S9}$$

$$P_{lv} = P_{lv,un} + E_{lv}\left(V_{lv} - V_{lv,un}\right) \tag{S10}$$

The aortic valve flow was given by:

$$Q_{ao} = \begin{cases} CQ_{ao} AR_{ao} \sqrt{P_{lv} - P_{sas}}, & if\ P_{lv} \ge P_{sas} \\ -CQ_{ao} AR_{ao} \sqrt{P_{sas} - P_{lv}}, & if\ P_{sas} < P_{lv} \end{cases} \tag{S11}$$

The ventricular elastance function was defined as:

$$E_{lv}(t) = E_{lv,min} + \frac{E_{lv,max} - E_{lv,min}}{2} e_v(t) \tag{S12}$$

$$e_v(t) = \begin{cases} 1 - cos\left(\frac{t}{T_{me}} \pi\right), & if\ 0 \le t < T_{me} \\ 1 + cos\left(\frac{t - T_{me}}{T_{ce} - T_{me}} \pi\right), & if\ T_{me} \le t < T_{ce} \\ 0, & if\ T_{ce} \le t < RR \end{cases} \tag{S13}$$

The aortic valve opening area ratio was:

$$AR_{ao} = \frac{(1 - cos\ \theta_{ao})^2}{(1 - cos\ \theta_{max})^2} \quad \text{(S14)}$$

S1.5. Right Atrium

The right atrium was modeled analogously to the left atrium [S3]:

$$\frac{dV_{ra}}{dt} = Q_{svn} - Q_{ti} \quad \text{(S15)}$$

$$P_{ra} = P_{ra,un} + E_{ra}\left(V_{ra} - V_{ra,un}\right) \quad \text{(S16)}$$

$$Q_{ti} = \begin{cases} CQ_{ti}AR_{ti}\sqrt{P_{ra} - P_{rv}}, & if\ P_{ra} \geq P_{rv} \\ -CQ_{ti}AR_{ti}\sqrt{P_{rv} - P_{ra}}, & if\ P_{ra} < P_{rv} \end{cases} \quad \text{(S17)}$$

$$E_{ra}(t) = E_{ra,min} + \frac{E_{ra,max} - E_{ra,min}}{2} e_a(t) \quad \text{(S18)}$$

$$AR_{ti} = \frac{(1 - cos\ \theta_{ti})^2}{(1 - cos\ \theta_{max})^2} \quad \text{(S19)}$$

S1.6. Right Ventricle

$$\frac{dV_{rv}}{dt} = Q_{ti} - Q_{po} \quad \text{(S20)}$$

$$P_{rv} = P_{rv,un} + E_{rv}\left(V_{rv} - V_{rv,un}\right) \quad \text{(S21)}$$

$$Q_{po} = \begin{cases} CQ_{po}AR_{po}\sqrt{P_{rv} - P_{pas}}, & if\ P_{rv} \geq P_{pas} \\ -CQ_{po}AR_{po}\sqrt{P_{pas} - P_{rv}}, & if\ P_{pas} < P_{rv} \end{cases} \quad \text{(S22)}$$

$$E_{rv}(t) = E_{rv,min} + \frac{E_{rv,max} - E_{rv,min}}{2} e_v(t) \quad \text{(S23)}$$

$$AR_{po} = \frac{\left(1 - cos\ \theta_{po}\right)^2}{(1 - cos\ \theta_{max})^2} \quad \text{(S24)}$$

S2. Vascular System Modeling

S2.1. Systemic Circulation

The systemic circulation was modeled using resistance–compliance–inertance (R–C–L) elements [S3].

Aortic Sinus

$$\begin{cases} \dfrac{dP_{sas}}{dt} = \dfrac{Q_{ao} - Q_{sas}}{C_{sas}} \\ \dfrac{dQ_{sas}}{dt} = \dfrac{P_{sas} - P_{sat} - R_{sas}Q_{sas}}{L_{sas}} \\ P_{sas} - P_{sas,un} = \dfrac{1}{C_{sas}}\left(V_{sas} - V_{sas,un}\right) \end{cases} \quad (S25)$$

Systemic Artery

$$\begin{cases} \dfrac{dP_{sat}}{dt} = \dfrac{Q_{sas} - Q_{sat}}{C_{sat}} \\ \dfrac{dQ_{sat}}{dt} = \dfrac{P_{sat} - P_{svn} - \left(R_{sat} + R_{sar} + R_{scp}\right)Q_{sat}}{L_{sat}} \\ P_{sat} - P_{sat,un} = \dfrac{1}{C_{sat}}\left(V_{sat} - V_{sat,un}\right) \end{cases} \quad (S26)$$

Systemic Vein

$$\begin{cases} \dfrac{dP_{svn}}{dt} = \dfrac{Q_{sat} - Q_{svn}}{C_{svn}} \\ Q_{svn} = \dfrac{P_{svn} - P_{ra}}{R_{svn}} \\ P_{svn} - P_{svn,un} = \dfrac{1}{C_{svn}}\left(V_{svn} - V_{svn,un}\right) \end{cases} \quad (S27)$$

S2.2. Pulmonary Circulation

Pulmonary Arterial Sinus

$$\begin{cases} \dfrac{dP_{pas}}{dt} = \dfrac{Q_{po} - Q_{pas}}{C_{pas}} \\ \dfrac{dQ_{pas}}{dt} = \dfrac{P_{pas} - P_{pat} - R_{pas}Q_{pas}}{L_{pas}} \\ P_{pas} - P_{pas,un} = \dfrac{1}{C_{pas}}\left(V_{pas} - V_{pas,un}\right) \end{cases} \quad (S28)$$

Pulmonary Artery

$$\begin{cases} \dfrac{dP_{pat}}{dt} = \dfrac{Q_{pas} - Q_{pat}}{C_{pat}} \\ \dfrac{dQ_{pat}}{dt} = \dfrac{P_{pat} - P_{pvn} - \left(R_{pat} + R_{par} + R_{pcp}\right)Q_{pat}}{L_{pat}} \\ P_{pat} - P_{pat,un} = \dfrac{1}{C_{pat}}\left(V_{pat} - V_{pat,un}\right) \end{cases} \quad (S29)$$

Pulmonary Vein

$$\begin{cases} \frac{dP_{pvn}}{dt} = \frac{Q_{pat} - Q_{pvn}}{C_{pvn}} \\ Q_{pvn} = \frac{P_{pvn} - P_{la}}{R_{pvn}} \\ P_{pvn} - P_{pvn,un} = \frac{1}{C_{pvn}} \left(V_{pvn} - V_{pvn,un}\right) \end{cases} \tag{S30}$$

Parameter Table:

Table S1: Heart Parameters

Parameter	Value
CQ_{ao}	350 ml/(s mmHg$^{0.5}$)
CQ_{mi}	400 ml/(s mmHg$^{0.5}$)
$E_{lv,max}$	2.5 mmHg/ml
$E_{lv,min}$	0.07 mmHg/ml
$P_{lv,un}$	1 mmHg
$V_{lv,un}$	5 ml
$E_{la,max}$	0.25 mmHg/ml
$E_{la,min}$	0.15 mmHg/ml
$P_{la,un}$	1 mmHg
$V_{la,un}$	4 ml
CQ_{po}	350 ml/(s mmHg$^{0.5}$)
CQ_{ti}	400 ml/(s mmHg$^{0.5}$)

https://doi.org/10.3390/xxxxx

$E_{rv,max}$	1.15 mmHg/ml
$E_{rv,min}$	0.07 mmHg/ml
$P_{rv,un}$	1 mmHg
$V_{rv,un}$	10 ml
$E_{ra,max}$	0.25 mmHg/ml
$E_{ra,min}$	0.15 mmHg/ml
$P_{ra,un}$	1 mmHg
$V_{ra,un}$	4 ml

Table S2: Systematic Circulation Parameters

**Parameter**	**Value**
C_{sas}	0.08 ml/mmHg
R_{sas}	0.003 mmHg s/ml
L_{sas}	0.000062 mmHg s^2/ml
C_{sat}	1.6 ml/mmHg
R_{sat}	0.05 mmHg s/ml
L_{sat}	0.0017 mmHg s^2/ml
R_{sar}	0.5 mmHg s/ml
R_{scp}	0.52 mmHg s/ml
R_{svn}	0.075 mmHg s/ml

C_{svn}	20.5 ml/mmHg

Table S3: Pulmonary Circulation Parameters

Parameter	Value
C_{pas}	0.18 ml/mmHg
R_{pas}	0.002 mmHg s/ml
L_{pas}	0.000052 mmHg s^2/ml
C_{pat}	3.8 ml/mmHg
R_{pat}	0.01 mmHg s/ml
L_{pat}	0.0017 mmHg s^2/ml
R_{par}	0.05 mmHg s/ml
R_{pcp}	0.07 mmHg s/ml
R_{pvn}	0.006 mmHg s/ml
C_{pvn}	20.5 ml/mmHg

Table S4: Valve Dynamics Parameters

Parameter	Value
$K_{p,mi}, K_{p,ao}, K_{p,ti}, K_{p,po}$	5500 ml/mmHg
$K_{f,mi}, K_{f,ao}, K_{f,ti}, K_{f,po}$	50 s^{-1}
$K_{b,mi}, K_{b,ao}, K_{b,ti}, K_{b,po}$	2 rad/(s ml)
$K_{v,mi}, K_{v,ti}$	3.5 rad/(s ml)

$K_{v,ao}, K_{v,po}$	7 rad/(s ml)
θ_{max}	5/12 π rad

Table S5: EMG Distribution

Rhythm condition	RR generation method	Parameter / statistic	Value	Unit	Description
AF	EMG distribution	μG	0.5	s	Mean of the correlated pink Gaussian component
AF	EMG distribution	σG	0.05	s	Standard deviation of the correlated pink Gaussian component
AF	EMG distribution	γ	6	s^{-1} / Hz	Rate parameter of the exponential component
AF	Resulting RR statistic	μ	0.67	s	Mean RR interval resulting from the EMG distribution
AF	Resulting RR statistic	σ	0.17	s	Standard deviation of the resulting RR interval
AF	Resulting RR statistic	cv	0.26	dimensionless	Coefficient of variation of the resulting RR interval
NSR	Pink-noise-based Gaussian RR variability	μ	0.8	s	Mean RR interval
NSR	Pink-noise-based	σ	0.056	s	Standard deviation of RR interval, calculated as μ×cv

https://doi.org/10.3390/xxxxx

	Gaussian RR variability				
NSR	Pink-noise-based Gaussian RR variability	cv	0.07	dimensionless	Coefficient of variation

References:

[S1] M. Sosnowski, B. Korzeniowska, P. W. Macfarlane, M. Tendera, "Relationship between R-R interval variation and left ventricular function in sinus rhythm and atrial fibrillation as estimated by means of heart rate variability fraction", *Cardiol J* **2011**, *18* (5), 538, https://doi.org/10.5603/cj.2011.0010.

[S2] M. Tanabe, K. Onishi, K. Dohi, T. Kitamura, M. Ito, T. Nobori, T. Nakano, "Assessment of left ventricular systolic function in patients with chronic atrial fibrillation and dilated cardiomyopathy using the ratio of preceding to prepreceding R-R intervals", *Int J Cardiol* **2006**, *108* (2), 197, https://doi.org/10.1016/j.ijcard.2005.05.001.

[S3] T. Korakianitis, Y. Shi, "Numerical simulation of cardiovascular dynamics with healthy and diseased heart valves", *J Biomech* **2006**, *39* (11), 1964, https://doi.org/10.1016/j.jbiomech.2005.06.016.
